# Effects of different surgical techniques and displacement distances on the soft tissue profile via orthodontic-orthognathic treatment of class II and class III malocclusions

**DOI:** 10.1186/s13005-021-00264-4

**Published:** 2021-04-14

**Authors:** Stephan Christian Möhlhenrich, Florian Kötter, Florian Peters, Kristian Kniha, Sachin Chhatwani, Gholamreza Danesh, Frank Hölzle, Ali Modabber

**Affiliations:** 1 Department of Orthodontics, University Witten/Herdecke, Alfred-Herrhausen-Straße 45, 58448 Witten, Germany; 2 Department of Oral and Maxillofacial Surgery, University Hospital of Aachen, Pauwelsstraße 30, 52074 Aachen, Germany

**Keywords:** Class II malocclusion, Class III malocclusion, Orthodontic camouflage, Orthognathic surgery, Soft tissue profile, Displacement distance

## Abstract

**Background:**

Orthognathic surgery can be carried out using isolated mandibular or maxillary movement and bimaxillary procedures. In cases of moderate skeletal malocclusion, camouflage treatment by premolar extraction is another treatment option. All these surgical procedures can have a different impact on the soft tissue profile.

**Methods:**

The changes in the soft tissue profile of 187 patients (Class II: 53, Class III: 134) were investigated. The treatment approaches were differentiated as follows: Class II: mandible advancement (MnA), bimaxillary surgery (MxS/MnA), upper extraction (UpEX), or Class III: maxillary advancement (MxA), mandible setback (MnS), bimaxillary surgery (MxA/MnS), and lower extraction (LowEX) as well as the extent of skeletal deviation (moderate Wits appraisal: − 7 mm to 7 mm, pronounced: Wits <− 7 mm, > 7 mm, respectively). This resulted in five groups for Class II treatment and seven groups for Class III treatment.

**Results:**

In the Class II patients, a statistically significant difference (*p* ≤ 0.05) between UpEX and moderate MnA was found for facial profile (N′-Prn-Pog’), soft tissue profile (N′-Sn-Pog’), and mentolabial angle (Pog’-B′-Li). In the Class III patients, a statistically significant differences (*p* ≤ 0.05) occurred between LowEX and moderate MxA for facial profile (N′-Prn-Pog’), soft tissue profile (N′-Sn-Pog’), upper and lower lip distacne to esthetic line (Ls/Li-E-line), and lower lip length (Sto-Gn’). Only isolated significant differences (*p* < 0.05) were recognized between the moderate surgical Class II and III treatments as well between the pronounced Class III surgeries. No statistical differences were noticed between moderate and pronounced orthognathic surgery.

**Conclusions:**

When surgery is required, the influence of orthognathic surgical techniques on the profile seems to be less significant. However, it must be carefully considered if orthognathic or camouflage treatment should be done in moderate malocclusions as a moderate mandibular advancement in Class II therapy will straighten the soft tissue profile much more by increasing the facial and soft tissue profile angle and reducing the mentolabial angle than camouflage treatment. In contrast, moderate maxillary advancement in Class III therapy led to a significantly more convex facial and soft tissue profile by decreasing distances of the lips to the E-Line as well as the lower lip length.

**Supplementary Information:**

The online version contains supplementary material available at 10.1186/s13005-021-00264-4.

## Introduction

Severe dentofacial skeletal malocclusions can usually be treated using a combined orthodontic and orthognathic treatment approach. The objective of orthognathic surgery is to correct these deformities to achieve an adequate occlusion in patients with severe skeletal Class II or Class III discrepancy after completion of development of the dentition by repositioning the maxilla, the mandible, or both. Additionally, and at least as important, facial deformities can be resolved close to functional malocclusion with orthognathic surgery.

Orthognathic surgery requires a complex interdisciplinary approach, which can be divided into different parts [1]. Usually, patients start with a preoperative orthodontic preparation, followed by planning for and the implementation of surgical adjustments, and postsurgical orthodontic treatment to achieve the final occlusion [2]. Presurgical orthodontic treatment aims to prevent dental decompensation that will enable a good surgical correction of the jaw discrepancy [2]. Postsurgical orthodontic treatment should to settle the dental arches and ensure precise tooth positioning [3]. However, in cases of moderate skeletal malocclusions, orthodontic treatment options are available that include extraction of the premolars for dental compensation in borderline cases of Class II or Class III patients that do not want surgery [4].

All surgical Class II and Class III treatments, including camouflage treatment by premolar extractions, have different effects on the facial outcome [5, 6]. This is important to know for the clinician, because the soft tissue profile and its components like lip position and facial convexity play an important role in facial esthetics [7–9]. Recently, Mousavi et al. reported, that a majority of test subjects preferred a lip position slightly anterior to the Ricketts norm in men but not in woman [10]. All groups favored profiles slightly less convex than the Legan-Burstone norm for men and women. However, they reported that female test subjects may have a wider zone of acceptability compared to males, in terms of facial convexity.

Regarding the amount of displacement distance, Eslami et al. recommended that in Class III patients, a Wits appraisal greater than − 5.8 mm would be effectively corrected using camouflage treatment; patients with a Wits appraisal less than − 5.8 mm must be treated by surgery [11]. Therefore, it is extremely important to understand the soft tissue changes caused by different surgical techniques and the extent of the segment movements.

Currently, there are no comparative studies about the effects of different orthognathic surgical methods on a patient’s soft tissue profile or on cases of moderate malocclusion of extraction therapy in Class II and Class III patients. However, in daily practice it is not uncommon to decide if camouflage treatment or orthognathic surgery would be better for the patient. Besides final occlusion and function, the soft tissue profile changes also need to be considered in the decision-making process.

This retrospective study focused on borderline patients and patients who clearly required orthognathic surgery without a solely orthodontic treatment alternative. The aim was to determine the influence of these different treatment approaches on the soft tissue profile. The first hypothesis of this study was that orthognathic treatment of moderate or pronounced skeletal Class II and Class III patients would lead to related changes in the soft tissue profile within each skeletal group. The second hypothesis was that camouflage treatment with premolar extraction within the moderate malocclusion group would have less of an influence on the soft tissue than orthodontic-orthognathic treatment.

## Materials & methods

Ethical approval to conduct this retrospective investigation was obtained from the institutional review board of the Ethics Commission of Rheinisch-Westfälische Technische Hochschule (RWTH) Aachen University, Germany (No 091/16). Relevant data, including lateral cephalograms, were collected from the patients that required combined orthodontic-orthognathic or camouflage treatment with premolar extraction for moderate or pronounced malocclusion treatment. All lateral cephalograms were obtained, as part of regular treatment planning and execution, by an experienced technician using a cephalometric imaging device (Orthophos SL 2D, Dentsply Sirona, Bensheim, Germany; Settings: 73 kV, 15 mAs, effective radiation 9.2 s) at the Department of Maxillofacial Surgery, University of Aachen, Germany. Routine calibration, system quality assurance, and imaging testing of the machine were checked at regular intervals. The examined subjects were aligned using optical localizers to the midsagittal plane and the Frankfurt horizontal plane to ensure symmetry.

The treatment approach was determined by considering the initial dental or skeletal findings, the possibilities and risks of orthodontic compensation or orthognathic surgery, and the patient’s subjective and objective feelings regarding facial aesthetics. Depending on the presurgical planning, premolar extraction, LeFort I osteotomy, a bilateral sagittal split osteotomy, or both were carried out.

This study included a total of 187 patients (101 females, 86 males). For inclusion in this study, skeletal growth had to be completed as detected on lateral cephalometric radiographs. For this purpose, the degree of maturity of the third cervical vertebra was determined based on the six stages according to Hassel and Farman [12]. Furthermore, in the surgical groups no operative corrections in transverse direction, such as lateral side shifting of the lower jaw were allowed. In addition, patients that had undergone previous surgeries that involved the middle or lower face, or additional procedures, such as genioplasty or rhinoplasty as well as patients with cleft lip and palate or other congenital craniofacial anomalies were excluded. In the camouflage groups the space closure was performed from anterior with fixed appliances and the support of Class II/III elastics or if necessary fixed Class II appliances. Herbst appliance or skeletal anchorage were not used. The corresponding demographic treatment data of all the patients are presented in Table 1.

**Table 1:** Patients’ demographic and treatment data after malocclusion and appropriate treatment

Malocclusion	Group	Treatment	N	Gender	Mean/Range of age (years)
Moderate class II	1	Mandibular advancement	18	11 females	29.6 (18.0–47.0)
				7 males	26.0 (19.0–40.0)
	2	Mandibular advancement / maxillary setback	10	7 females	28.8 (17.0–41.0)
				3 males	27.0 (25.0–29.0)
	3	Upper premolar extraction	11	6 females	17.7 (15.0–24.0)
				5 males	16.8 (16.0–17.0)
Moderate class III	4	Mandibular setback	6	3 females	27.2 (18.0–34.0)
				3 males	26.0 (20.0–31.0)
	5	Maxillary advancement	8	4 females	32.5 (19.0–51.0)
				4 males	29.8 (26.0–33.0)
	6	Mandibular setback / maxillary advancement	15	8 females	25.6 (18.0–38.0)
				7 males	23.0 (17.0–33.0)
	7	Lower premolar extraction	12	8 females	19.5 (15.0–36.0)
				4 males	18.0 (16.0–20.0)
Pronounced class II	1	Mandibular advancement	11	10 females	33.2 (20.0–40.0)
				1 male	33.0 (33.0–33.0)
	2	Mandibular advancement / maxillary setback	3	1 female	36.0 (36.0–36.0)
				2 males	27.0 (22.0–32.0)
Pronounced class III	4	Mandibular setback	19	12 females	26.8 (19.0–36.0)
				7 males	25.7 (19.0–35.0)
	5	Maxillary advancement	26	12 females	28.6 (17.0–47.0)
				14 males	28.6 (18.0–59.0)
	6	Mandibular setback / maxillary advancement	48	19 females	20.6 (16.0–28.0)
				29 males	25.5 (18.0–47.0)

The patient population was divided into groups according to the underlying skeletal Class (Class II, Class III) and the extent of the malocclusion (moderate, pronounced) as well as the type of therapy (monomaxillary, bimaxillary surgery, premolar extraction). The Wits appraisal value for a moderate skeletal Class II was considered to be up to 7 mm; for a Class III, that value is up to − 7 mm. All the values greater than that were regarded as pronounced malocclusions. The following groups were established for each of the skeletal classes:

### Class II patients

- Group 1 (*N* = 18): monomaxillary surgery (moderate mandibular advancement)
- Group 2 (*N* = 11): monomaxillary surgery (pronounced mandibular advancement)
- Group 3 (*N* = 10): bimaxillary surgery (moderate mandibular advancement and maxillary setback)
- Group 4 (*N* = 3): bimaxillary surgery (pronounced mandibular advancement and maxillary setback)
- Group 5 (*N* = 11): camouflage treatment (upper premolar extraction)

### Class III patients

- Group 1 (*N* = 8): monomaxillary surgery (moderate maxillary advancement)
- Group 2 (*N* = 26): monomaxillary surgery (pronounced maxillary advancement)
- Group 3 (*N* = 6): monomaxillary surgery (moderate mandibular setback)
- Group 4 (*N* = 19): monomaxillary surgery (pronounced mandibular setback)
- Group 5 (*N* = 15)): bimaxillary surgery (moderate mandibular setback and maxillary advancement)
- Group 6 (*N* = 48): bimaxillary surgery (pronounced mandibular setback and maxillary advancement)
- Group 7 (*N* = 12): camouflage treatment (lower premolar extraction)

A total of 374 lateral cephalograms were evaluated, two cephalograms from each patient before treatment (T0) and after treatment (T1). The differences T∆ (T1-T0) between the treatment results were calculated to determine the extent of the hard and soft tissue change.

**Fig. 1:**
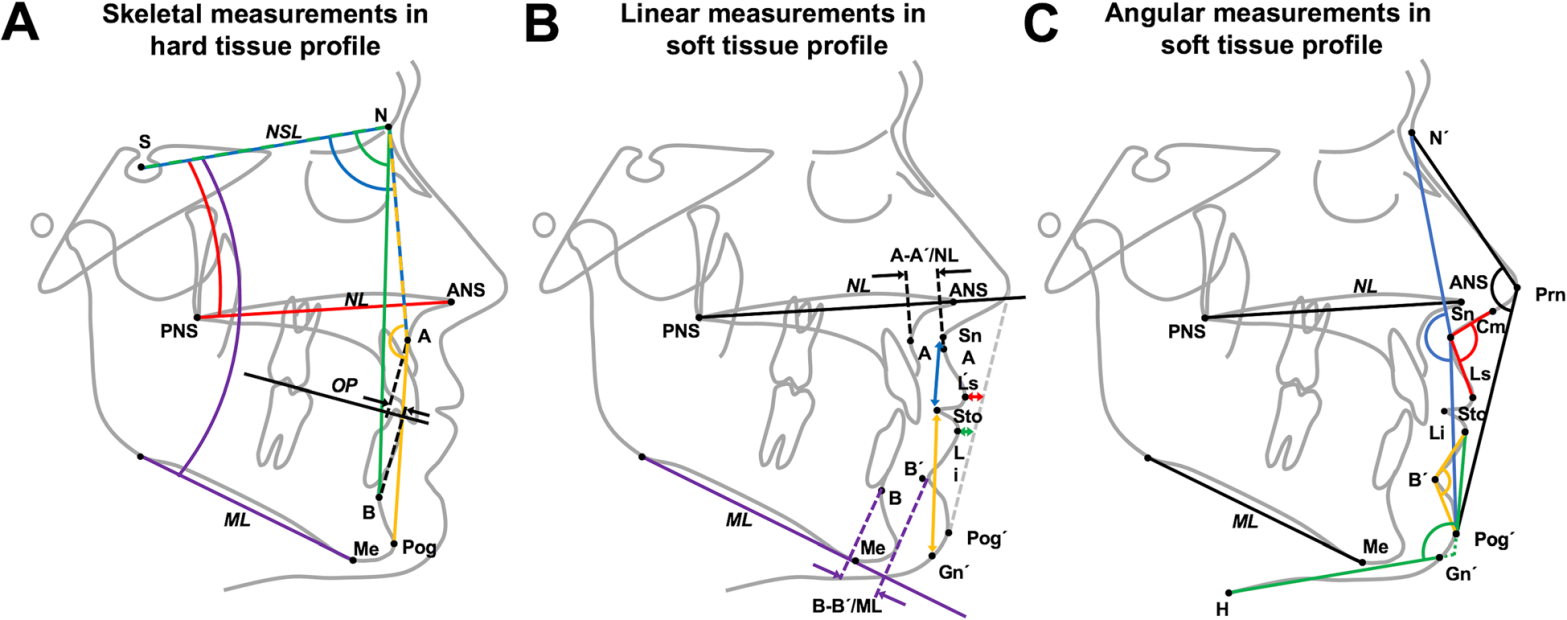
A) Angular skeletal measurements: SNA (blue); SNB (green); maxillary inclination, NL-NSL (red); mandibular inclination, ML-NSL (purple); skeletal profile, N-A-Pog (yellow). B) Linear measurements in the soft tissue profile: upper lip length, Sn-Sto (blue), upper lip thickness, A–A’ on NL (black), upper lip to Esthetic line, Ls-E-line (red), lower lip length, Sto-Gn’ (yellow), upper lip thickness, B–B′ on ML (purple), lower lip to esthetic line, Li-E-line (green). C) Angular measurements in the soft tissue profile*:* facial profile, N′-Prn-Pog’ (black), soft tissue profile, N′-Sn-Pog’ (blue), nasolabial angle, Cm-Sn-Ls (red), mentolabial angle, Pog’-B′-Li (Yellow), lip-chin-throat angle, LiPog’-Gn’H (green)

The cephalometric analysis was performed by one experienced operator using software support (OnyxCeph v3 LAB, Image Instruments GmbH, Chemnitz, Germany). The linear and angular measurements were made according to Kinzinger et al. [13] as well as Ghassemi et al. [5, 6] and documented to measure the skeletal changes of the maxilla and mandible as well as the soft tissue profile (Fig. 1):

- *Skeletal measurements:* SNA, SNB, Wits appraisal, maxillary inclination (NL-NSL), mandibular inclination (ML-NSL), skeletal profile (N-A-Pog)
- *Soft tissue measurements:* facial profile (N′-Prn-Pog’), soft tissue profile (N′-Sn-Pog’), nasolabial angle (Cm-Sn-Ls), mentolabial angle (Pog’-B′-Li), lip-chin-throat angle (LiPog’-Gn’H), upper lip length (Sn-Sto), upper lip thickness (A–A’ on NL), upper lip to esthetic line (Ls-E-line), lower lip length (Sto- Gn’), lower lip thickness (B–B′ on ML), and lower lip to esthetic line (Li-E-line)

### Statistical analysis

The statistical analysis between the groups was performed with GraphPad Prism V9 (GraphPad Software Inc., San Diego, CA, USA). Normal distribution was tested by Shapiro-Wilk tests. Because of partial insufficient normal distributions as well as the partly small sample size, non-parametric Mann-Whitney tests were applied for data analysis. The level of significance was set *p* ≤ 0.05. All results are expressed as mean values and (±) standard deviation (SD).

## Results

**Fig. 2:**
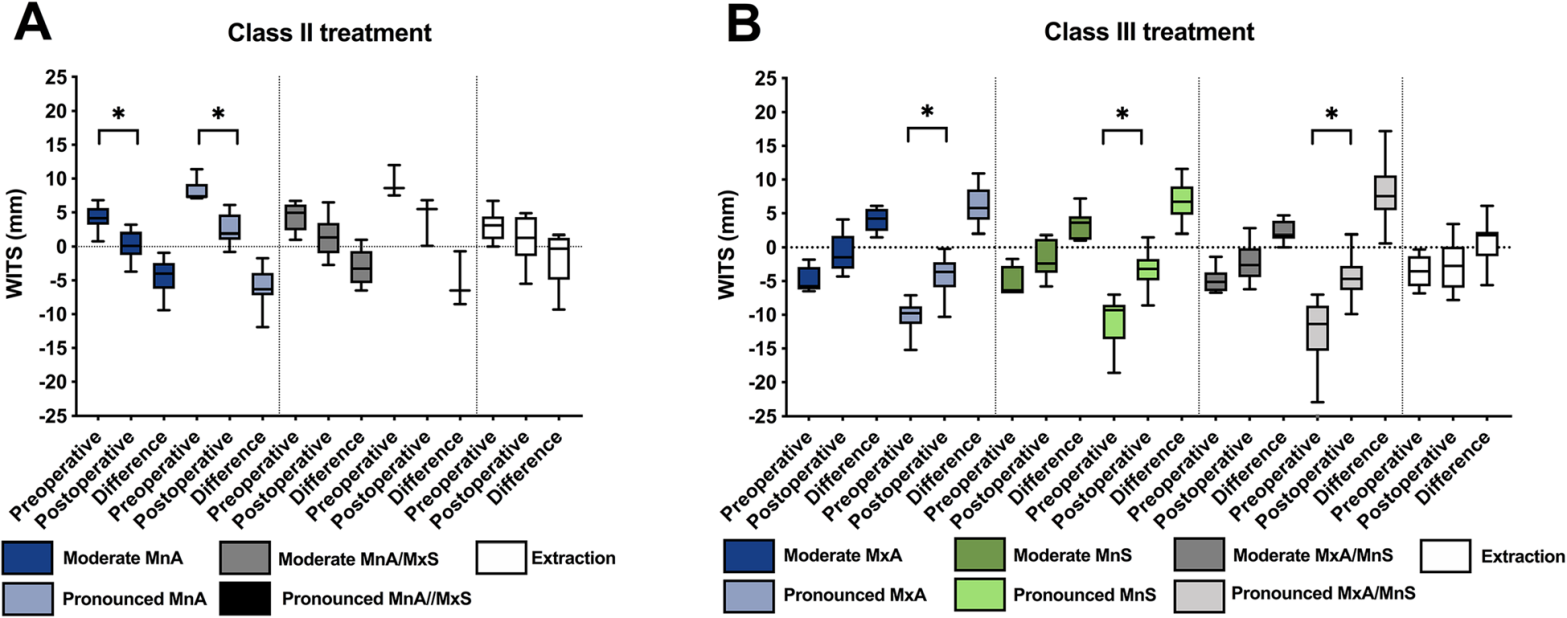
Box plots of the Wits changes (mm) in the moderate and pronounced Class II patients (A) and Class III patients (B) before and after monomaxillary surgery, bimaxillary surgery, and premolar extractions. MnA: mandibular advancement, MnS: mandibular setback, MxA: maxillary advancement, MxS: maxillary setback. Statistical significance: *P* < 0.05

**Fig. 3:**
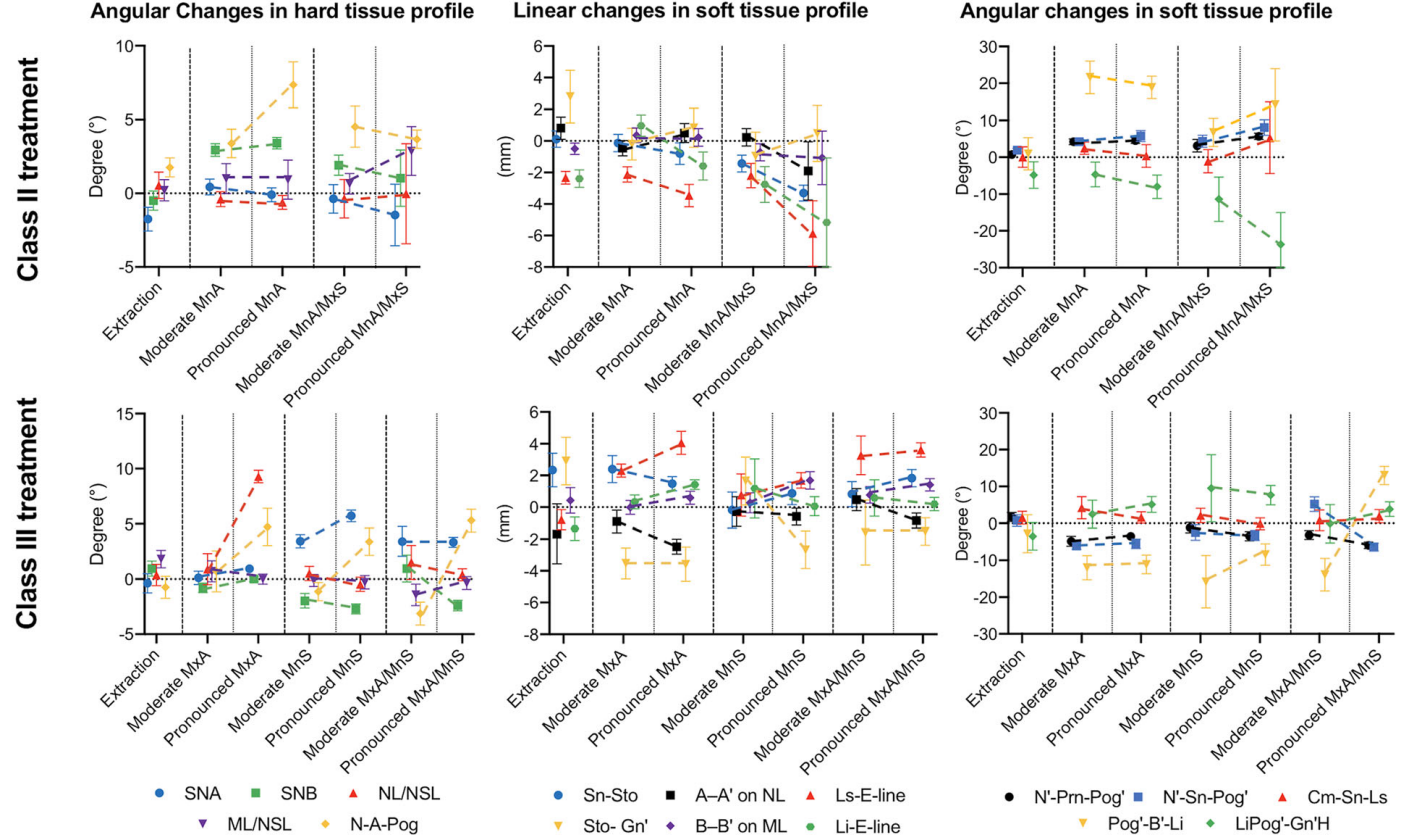
Interleaved mean values with the related standard error of mean (SEM) of the measured angular and linear changes in the hard and soft tissue profiles in moderate and pronounced Class II and Class III patients after monomaxillary surgery, bimaxillary surgery, and premolar extractions. MnA: mandibular advancement, MnS: mandibular setback, MxA: maxillary advancement, MxS: maxillary setback. Angular skeletal measurements: SNA (blue); SNB (green); maxillary inclination, NL-NSL (red); mandibular inclination, ML-NSL (purple); skeletal profile, N-A-Pog (yellow). Linear measurements in the soft tissue profile: upper lip length, Sn-Sto (blue), upper lip thickness, A–A’ on NL (black), upper lip to esthetic line, Ls-E-line (red), upper lip length, Sto- Gn’ (yellow), upper lip thickness, B–B′ on ML (purple), lower lip to esthetic line, Li-E-line (green). Angular measurements in the soft tissue profile*:* facial profile, N′-Prn-Pog’ (black), soft tissue profile, N′-Sn-Pog’ (blue), nasolabial angle, Cm-Sn-Ls (red), mentolabial angle, Pog’-B′-Li (Yellow), lip-chin-throat angle, LiPog’-Gn’H (green)

The changes in the Wits appraisal value depending on the surgical technique are illustrated in the box plots in Fig. 2. Mean values and SD of the measured angular and linear changes in the hard and soft tissue profiles as are shown in Tables 2 and 3. The mean values with the related standard error of mean (SEM) are presented in Fig. 3. The *p*-values of the significant differences of the corresponding statistical comparisons are demonstrated in Table 4. These mean values and SD of the measured parameters depending on the main surgical techniques regardless of the amount of displacement but with gender related corresponding p-values are shown in Table S1 (Supplementary materials). No statistical differences were found regarding the Wits appraisal values before treatment in the moderate Class II and Class III patients and the extraction patients (Fig. 2). Additionally, no differences were observed in the pronounced Class II and Class III patients; however, differences were observed between the pronounced Class II and Class III patients and the extraction patients (moderate malocclusion). Thus, the initial situations are generally comparable for moderate and pronounced malocclusions. Furthermore, in the Class II patients, the treatment resulted in statistically significant changes in Group 1 and Group 2, but not in Group 3, Group 4, and Group 5. In contrast, in the Class III patients, the treatment only resulted in statistically significant changes in Group 2, Group 4, and Group 6, but not Group 1, Group 3, Group 5, and Group 7.

**Table 2:** Mean values and standard deviation (SD) of the angular and linear changes T∆ (T1-T0) of the hard and soft tissue profiles in Class II patients depending on the extent of malocclusion and different surgical treatment techniques

Parameter	Class II malocclusion				
	Upper premolar extraction	Moderate MnA	Pronounced MnA	Moderate MxS/MnA	Pronounced MxS/MnA
	Mean (SD)	Mean (SD)	Mean (SD)	Mean (SD)	Mean (SD)
**SNA**	−1.75 (2.69)	0.43 (2.28)	−0.10 (1.56)	−0.37 (3.21)	− 1.47 (3.61)
**SNB**	−0.49 (2.15)	2.94 (1.81)	3.41 (1.29)	1.91 (2.27)	1.03 (3.32)
**WITS**	−2.04 (3.77)	−4.36 (2.25)	−6.07 (2.70)	−3.07 (2.60)	−5.23 (4.05)
**NL/NSL**	0.55 (2.97)	−0.39 (2.13)	−0.61 (1.56)	− 0.35 (4.32)	−0.03 (5.88)
**ML/NSL**	0.21 (2.39)	1.00 (4.34)	0.93 (4.41)	0.65 (2.30)	2.87 (2.87)
**N-A-Pog**	1.76 (2.14)	3.39 (4.11)	7.35 (5.15)	4.52 (4.60)	3.67 (1.08)
**N′-Prn-Pog’**	0.71 (3.78)	4.20 (3.23)	4.55 (2.56)	3.14 (5.63)	5.67 (1.50)
**N′-Sn-Pog’**	1.92 (1.69)	4.23 (3.00)	5.75 (4.90)	4.33 (5.39)	8.07 (3.66)
**Cm-Sn-Ls**	0.03 (9.20)	2.48 (6.99)	0.36 (10.20)	−1.07 (10.50)	5.27 (16.80)
**Pog’-B′-Li**	0.93 (14.50)	21.60 (18.80)	18.90 (10.10)	6.73 (12.70)	14.20 (16.90)
**LiPog’-Gn’H**	−4.83 (11.90)	−4.69 (14.20)	−7.99 (10.50)	− 11.40 (19.90)	−23.70 (15.00)
**Sn-Sto**	0.12 (1.72)	−0.13 (2.32)	−0.80 (2.26)	−1.43 (1.77)	−3.30 (0.87)
**A–A’ on NL**	0.81 (2.34)	−0.46 (2.09)	0.50 (1.99)	0.23 (1.82)	−1.90 (3.20)
**Ls-E-line**	−2.33 (1.34)	−2.12 (2.03)	−3.46 (2.33)	− 2.20 (2.55)	− 5.87 (3.59)
**Sto- Gn’**	2.80 (5.51)	−0.20 (4.26)	0.84 (4.10)	−0.96 (5.04)	0.47 (3.06)
**B–B′ on ML**	−0.49 (1.21)	0.36 (2.00)	0.23 (1.84)	− 0.72 (1.78)	−1.07 (2.94)
**Li-E-line**	−2.39 (1.84)	−0.97 (2.84)	−1.59 (2.94)	− 2.76 (3.78)	−5.17 (7.10)

**Table 3:** Mean values and standard deviation (SD) of the angular and linear changes T∆ (T1-T0) of the hard and soft tissue profiles in Class III patients depending on the extent of malocclusion and different surgical treatment techniques

Parameter	Class III malocclusion						
	Lower premolar extraction	Moderate MxA	Pronounced MxA	Moderate MnS	Pronounced MnS	Moderate MxA/MnS	Pronounced MxA/MnS
	Mean (SD)	Mean (SD)	Mean (SD)	Mean (SD)	Mean (SD)	Mean (SD)	Mean (SD)
**SNA**	−0.38 (3.04)	0.12 (1.73)	0.94 (1.74)	3.44 (1.46)	5.72 (2.29)	3.41 (5.20)	3.34 (3.16)
**SNB**	−0.96 (2.35)	−0.79 (1.24)	0.00 (1.15)	−1.97 (1.61)	−2.71 (1.99)	0.96 (4.71)	−2.40 (3.31)
**WITS**	0.78 (3.26)	3.95 (1.73)	6.22 (2.69)	3.38 (2.25)	7.03 (2.66)	3.14 (2.28)	8.35 (4.56)
**NL/NSL**	0.38 (3.35)	0.90 (3.97)	0.29 (2.90)	0.52 (1.56)	−0.47 (2.81)	1.52 (5.97)	0.40 (3.71)
**ML/NSL**	1.82 (2.75)	0.71 (2.68)	0.00 (2.37)	−0.12 (1.35)	−0.29 (2.65)	−1.43 (3.76)	− 0.36 (4.01)
**N-A-Pog**	−0.74 (3.46)	0.70 (5.27)	4.72 (8.59)	−1.13 (2.03)	3.37 (5.38)	−3.13 (4.02)	5.30 (7.09)
**N′-Prn-Pog’**	1.70 (4.32)	−4.84 (3.81)	− 3.47 (5.21)	−1.12 (3.45)	− 3.45 (5.31)	−3.13 (4.58)	−5.91 (6.04)
**N′-Sn-Pog’**	0.76 (5.45)	−6.09 (2.58)	− 5.50 (6.53)	−2.43 (5.08)	−3.24 (5.87)	− 5.20 (7.74)	− 6.36 (7.29)
**Cm-Sn-Ls**	1.53 (6.06)	4.23 (8.51)	1.61 (7.81)	2.13 (4.78)	−0.08 (6.88)	0.85 (10.70)	1.93 (12.60)
**Pog’-B′-Li**	−2.80 (17.80)	−12.00 (9.25)	−11.10 (12.90)	− 15.80 (17.50)	−8.42 (12.60)	−13.90 (17.10)	− 13.00 (17.00)
**LiPog’-Gn’H**	−3.49 (12.90)	2.49 (10.70)	5.14 (11.00)	9.52 (22.30)	7.66 (11.50)	−0.09 (20.10)	3.84 (13.60)
**Sn-Sto**	2.34 (3.68)	2.40 (2.41)	1.48 (2.28)	−0.18 (2.78)	0.87 (3.09)	0.82 (3.04)	1.83 (3.82)
**A–A’ on NL**	−1.68 (6.53)	−0.90 (2.01)	−2.48 (2.38)	−0.28 (2.27)	− 0.59 (2.33)	0.47 (2.69)	− 0.84 (3.29)
**Ls-E-line**	−0.78 (2.16)	2.31 (1.16)	4.06 (3.68)	0.77 (3.25)	1.69 (2.12)	3.27 (4.70)	3.61 (3.13)
**Sto- Gn’**	2.91 (5.18)	−3.53 (2.76)	−3.58 (5.49)	1.65 (3.70)	−2.67 (5.15)	−1.59 (7.90)	−1.52 (5.94)
**B–B′ on ML**	0.43 (2.80)	−0.01 (1.24)	0.59 (2.08)	0.28 (1.54)	1.69 (2.39)	0.77 (2.01)	1.41 (2.69)
**Li-E-line**	−1.35 (2.51)	0.33 (1.25)	1.42 (1.55)	1.18 (4.55)	0.06 (2.63)	0.58 (4.41)	0.20 (2.90)

**Table 4:** p-Values of the comparisons between the different treatment methods depending on the initial WITS value for Class II and III malocclusion

Malocclusion		Comparison			SNA	SNB	WITS	NL/NSL	ML/NSL	N-A-Pog	N′-Prn-Pog’	N′-Sn-Pog’	Cm-Sn-Ls	Pog’-B′-Li	LiPog’-Gn’H	Sn-Sto	A–A’ on NL	Ls-E-line	Sto- Gn’	B–B′ on ML	Li-E-line
**Class II**	Moderate	UpEx	vs	MnA	≤0.05^a^	≤0.001^a^	n.s.	n.s.	n.s.	n.s.	≤0.05^a^	≤0.05^a^	n.s.	≤0.01^a^	n.s.	n.s.	n.s.	n.s.	n.s.	n.s.	n.s.
			vs	MxS/MnA	n.s.	≤0.05^a^	n.s.	n.s.	n.s.	n.s.	n.s.	n.s.	n.s.	n.s.	n.s.	≤0.05^a^	n.s.	n.s.	n.s.	n.s.	n.s.
		MnA	vs	MxS/MnA	n.s.	n.s.	n.s.	n.s.	n.s.	n.s.	n.s.	n.s.	n.s.	≤0.05^a^	n.s.	n.s.	n.s.	n.s.	n.s.	n.s.	n.s.
	Pronounced	MnA	vs	MxS/MnA	n.s.	n.s.	n.s.	n.s.	n.s.	n.s.	n.s.	n.s.	n.s.	n.s.	n.s.	n.s.	n.s.	n.s.	n.s.	n.s.	n.s.
	MnA	Moderate	vs	Pronounced	n.s.	n.s.	n.s.	n.s.	n.s.	≤0.05^a^	n.s.	n.s.	n.s.	n.s.	n.s.	n.s.	n.s.	n.s.	n.s.	n.s.	n.s.
	MxS/MnA	Moderate	vs	Pronounced	n.s.	n.s.	n.s.	n.s.	n.s.	n.s.	n.s.	n.s.	n.s.	n.s.	n.s.	n.s.	n.s.	n.s.	n.s.	n.s.	n.s.
**Class III**	Moderate	LowEx	vs	MxA	≤0.01^a^	n.s.	≤0.01^a^	n.s.	n.s.	n.s.	≤0.01^a^	≤0.001^a^	n.s.	≤0.05^a^	n.s.	n.s.	n.s.	≤0.01^a^	≤0.01^a^	n.s.	≤0.05^a^
			vs	MnS	n.s.	n.s.	n.s.	n.s.	≤0.05^a^	n.s.	n.s.	n.s.	n.s.	n.s.	n.s.	n.s.	n.s.	n.s.	n.s.	n.s.	n.s.
			vs	MxA/MnS	≤0.05^a^	n.s.	n.s.	n.s.	≤0.05^a^	n.s.	≤0.01^a^	n.s.	n.s.	≤0.05^a^	n.s.	n.s.	n.s.	≤0.01^a^	n.s.	n.s.	n.s.
		MxA	vs	MnS	≤0.01^a^	n.s.	n.s.	n.s.	n.s.	n.s.	n.s.	n.s.	n.s.	n.s.	n.s.	n.s.	n.s.	n.s.	≤0.05^a^	n.s.	n.s.
			vs	MxA/MnS	n.s.	n.s.	n.s.	n.s.	n.s.	n.s.	n.s.	n.s.	n.s.	n.s.	n.s.	n.s.	n.s.	n.s.	n.s.	n.s.	n.s.
		MnS	vs	MxA/MnS	≤0.05^a^	n.s.	n.s.	n.s.	n.s.	n.s.	n.s.	n.s.	n.s.	n.s.	n.s.	n.s.	n.s.	n.s.	n.s.	n.s.	n.s.
	Pronounced	MxA	vs	MnS	≤0.001^a^	≤0.001^a^	n.s.	n.s.	n.s.	n.s.	n.s.	n.s.	n.s.	n.s.	n.s.	n.s.	≤0.01^a^	≤0.05^a^	n.s.	n.s.	n.s.
			vs	MxA/MnS	≤0.001^a^	≤0.001^a^	≤0.05^a^	n.s.	n.s.	n.s.	n.s.	n.s.	n.s.	n.s.	n.s.	n.s.	≤0.05^a^	n.s.	n.s.	n.s.	≤ 0.05^a^
		MnS	vs	MxA/MnS	≤0.001^a^	n.s.	n.s.	n.s.	n.s.	n.s.	n.s.	n.s.	n.s.	n.s.	n.s.	n.s.	n.s.	≤0.05^a^	n.s.	n.s.	n.s.
	MxA	Moderate	vs	Pronounced	≤0.01^a^	n.s.	≤0.05^a^	n.s.	n.s.	n.s.	n.s.	n.s.	n.s.	n.s.	n.s.	n.s.	n.s.	n.s.	n.s.	n.s.	n.s.
	MnS	Moderate	vs	Pronounced	n.s.	n.s.	≤0.01^a^	n.s.	n.s.	≤0.05^a^	n.s.	n.s.	n.s.	n.s.	n.s.	n.s.	n.s.	n.s.	≤0.05^a^	n.s.	n.s.
	MxA/MnS	Moderate	vs	Pronounced	n.s.	≤0.05^a^	≤0.001^a^	n.s.	n.s.	≤0.001^a^	n.s.	n.s.	n.s.	n.s.	n.s.	n.s.	n.s.	n.s.	n.s.	n.s.	n.s.

*UpEx* Upper premolar extraction, *LorEx* Lower premolar extraction, *MnA* Mandibular advancement, *MnS* Mandibular setback, *MxA* Maxillary advancement, *MxS* Maxillary setback. ^a^ Statistically significant

Although the skeletal differences between the treatment groups were partially statistically significant, especially the SNA, SNB and WITS, the corresponding changes in the soft tissue profile were only sporadically statistically significant. Therefore, the observed differences are subjective rather than statistical and thus mainly descriptive.

Extraction therapy has an effect on the soft tissue profile, regardless of whether Class II or Class III treatment is administered. The extent of the profile change in the camouflage groups was comparable to the changes in the moderate surgical groups in Class II with bimaxillary surgery and Class III patients with mandibular setback. However, a statistically significant difference (*p* < 0.05) in the profile changes between premolar extraction and moderate mandibular advancement was found in the Class II patients for N′-Prn-Pog’, N′-Sn-Pog ‘and Pog’-B’-Li, and between extraction and moderate maxillary advancement in the Class III patients for N’-Prn-Pog’, N’-Sn-Pog’, Pog’-B’-Li, Ls-E-line, Li-E-line and Sto- Gn’ (Table 4). Additionally, significant differences were noticed between lower premolar extraction and moderate bimaxillary surgery for N′-Prn-Pog’, Pog’-B′-Li and, Ls-E-line.

No statistical differences of the soft tissue changes were determined in the comparisons between the moderate and pronounced malocclusions regardless of their classification or underlying surgical treatment concept, although an enhancement of the respective soft tissue change was at least descriptively recognizable (Tables 2, 3).

Within the Class II as well as Class III groups with orthognathic surgical treatment only isolated significant differences are found (Class II: Pog’-B′-Li, MnA: 21.60 ± 18.80° vs. MxS/MnA: 6.73 ± 12.70°, *p* < 0.05; Class III, Sto- Gn’, MxA: − 3.53 ± 2.76 mm vs. MnS: 1.65 ± 3.70 mm, p < 0.05). Otherwise, there were no significant differences.

With regard to the surgical treatment of the pronounced malocclusions, no significant differences were found in Class II therapy, but in Class III treatment for A-A’ on NL (MxA: − 2.48 ± 2.38 mm vs MnS: − 0.59 ± 2.33 mm, *p* < 0.01; MxA: − 2.48 ± 2.38 mm vs. MxA/MnS: − 0.84 ± 3.29 mm, p < 0.05) and Ls-E-line. (MxA: 4.06 ± 3.68 mm vs MnS: 1.69 ± 2.12 mm, p < 0.05; MnS: 1.69 ± 2.12 mm vs. MxA/MnS: 3.61 ± 3.13 mm, p < 0.05).

## Discussion

Different aesthetic facial units with corresponding subunits of soft tissue profile are known, which include forehead, nose, eyes, cheek, upper and lower lip, chin, ear and the neck [14]. In this context, orthognathic surgery is able to particularly influence nose, lips, chin, and cervical length [5, 6, 15–23]. Additionally, camouflage treatment of moderate skeletal malocclusion by premolar extraction could also have an impact on the soft tissue profile of Class II and III patients [13, 24, 25]. Therefore, it is important for the patient and the clinician to know about the different influences of treatment concepts on the soft tissue profile, as this may have an impact on the choice of therapy. Accordingly, the soft tissue profile changes of 187 patients (Class II: 53, Class III: 134), depending on different therapy concepts as well as the extent of the malocclusion according to the Wits appraisal, were studied retrospectively.

However, this investigation is limited in its power by the sample size number and consequently the partly inhomogeneous group distribution, respectively. This is due to the large number of groups and simultaneously too few subjects in some groups. Furthermore, a sizeable part of the collected data was not normally distributed and non-parametric tests had to be applied. A detailed gender-specific classification and analysis was not possible for corresponding individual subgroups.

Another aspect to be discussed with regard to the study design is the present classification of “moderate” or “pronounced” malocclusion. In the present investigation, camouflage treatment was performed in Class II patients up to a Wits appraisal of 6.7 mm and in the Class III patients of − 6.8 mm. Therefore, the corresponding borderline values of the WITS appraisals were set about + 7 mm in Class II and − 7 mm in Class III patients. Thus, this threshold value is higher than already postulated in the literature. Ghassemi et al. distinguished surgical Class III therapies between patients who received maxillary advancement less than 6 mm and those with 6 mm or more, and patients who had mandible setback less than 5 mm and those with 5 mm or more [5, 6]. However, no information about the corresponding Wits appraisal was provided. With regard to camouflage treatment in Class III patients, Eslami et al. reported that patients with Wits appraisal greater than − 5.8 mm could be treated successfully by camouflage, while those with Wits appraisal less than − 5.8 mm are advised to be treated surgically [11]. Recently, Raposo et al. reported in a systematic review and meta-analysis the effects of orthodontic camouflage and orthodontic-orthognathic surgical treatment in Class II malocclusion that it was not possible to use the Wits variable in their work, since only two studies focused on this topic [26, 27]. These studies compared the treatment effect of camouflage treatment using the Herbst appliance and orthognathic surgery and reported corresponding Wits appraisals between 2.55 ± 2.06 mm and 4.72 ± 3.01 mm [26] as well as 2.13 ± 1.96 mm and 3.64 ± 2.65 mm [27]. In contrast, in the current investigation the mean Wits value before treatment in the camouflage group in Class II patients was about 3.04 ± 2.18 mm and in Class III patients about − 3,58 ± 2,35 mm, while the values in the corresponding surgical groups ranged for moderate Class II between 4.21 ± 1.76 mm and 4.45 ± 2.02 mm, as well as for Class III between − 4.84 ± 1.82 mm and − 5.18 ± 2.22 mm. This means that the present values are comparatively slightly higher to those reported in the literature, at least for Class II patients.

In the systematic review by Raposo et al. it was found that the surgical-orthodontic treatment in Class II malocclusion was more effective than the orthodontic camouflage treatment with regard to skeletal outcome as well as soft tissue profile including the nose (N′-Prn-Pog’) [25]. However, they concluded that camouflage treatment may represent an alternative to surgical-orthodontic treatment, especially with regard to the Li–E-line, skeletal profile (N–A–Pog), and soft tissue profile (N′-Sn-Pog’) measurements. But they also indicated that, for the majority of the measurements, especially dental ones, patients undergoing surgical-orthodontic treatment presented a more severe pretreatment condition, so the study’s conclusions should be interpreted with caution. In this context, Kinzinger et al. reported on the camouflage treatment in Class II patients by upper premolar extraction that significant reductions in profile convexity were achievable only by combined orthodontic and surgical treatment of the malocclusion [13]. Furthermore, when performing camouflage treatment combined with maxillary premolar extractions in adults, an increase in the nasolabial angle, which is often esthetically undesirable, has to be discussed as a potential side effect and it has to be considered when selecting the appropriate therapeutic approach.

Rabie et al. investigated the differences in the morphological characteristics of borderline Class III patients who had undergone camouflage orthodontic treatment or orthognathic surgery, and they compared the treatment effects of both therapeutic options [28]. They reported that both treatment techniques led to changes in the lower dental-alveolus and lower incisors. They also found that the most pronounced soft tissue changes were limited to the lower lip rather than both lips, regardless of the treatment group. This finding was different from what was reported in another study, which described a change in both lips [29].

Different studies have suggested that bimaxillary surgery should be considered in the treatment of Class III malocclusions because the additional maxillary advancement will reduce the need for extensive mandibular setback [15]. This is justified by the significant increase in the width of the airway postoperatively in comparison to the single mandibular setback [16] or the reduced risk of an undesired effect in the submental region, which may require additional operations, such as liposuction [5]. Furthermore, double jaw surgery should provide better long-term stability [30].

Generally, the present investigation demonstrated that, in both of the surgical Class II groups with an increasing displacement distance, the distance between the lips and the E-line decreased (Ls-E-line, Li-E-line), both lips become thinner (A–A’ on NL, B–B′ on ML), while the upper lip (Sn-Sto) became shorter and the lower lip (Sto- Gn’) became longer. However, these changes in the profile are not statistically significant. This is probably due to the large variability of post-treatment lip positions [19]. In this context, in terms of flattening, the mentolabial angle (Pog’-B′-Li) is more affected by mandibular advancement than by bimaxillary procedures. Furthermore, it was particularly noticeable that the lip-chin-throat angle (LiPog’-Gn’H) became smaller with increasing displacement, especially with the bimaxillary procedures. This information can be important with regard to the possibly simultaneous aesthetic treatment of a double chin.

In the Class III groups, the changes in the soft tissue profile depending on the surgical technique or the amount of displacement differed. It was found that the soft tissue behavior differed between the maxillary advancements and the mandibular setback or bimaxillary procedures, which were more equal to each other. Especially, the distance between the upper lip and the E-line increased with increasing displacement distance for the maxillary advancement or bimaxillary procedures, while the decrease in the lower lip was comparatively low after mandible setback or bimaxillary surgery. Linear measurements seemed to be more affected by maxillary advancement than angular findings. The surgical intervention led to a shortening and thickening of the lower lip length, but a slight extension and thinning of the upper lip. This behavior is most often found in the context of maxillary advancement. Regarding the angular changes, it can be seen that the mentolabial fold becomes more noticeable and the lip-chin-throat angle increases. While this primarily applies to maxillary advancement, it is also seen in mandibular setback, which is important with regard to the development of a double chin. In cases of an already existing double chin, this should be considered when deciding on the appropriate surgical approach.

Regarding statistically significant changes, differences between the profile changes were found more frequently for skeletal than for the soft tissue measurements, this is already known from other studies, which reported that the soft tissue is relatively less displaceable than the underlying hard tissue [19, 23]. In the present study, there were no significant differences between the angular and linear changes (T∆: T1-T0) of moderate and pronounced malocclusion, significant differences between camouflage therapy and orthognathic surgery were already found within the moderate Class II and III treatment. These differences are primarily due to changes caused by surgical procedures, while the camouflage therapy caused only minor changes.

Thus, mandibular advancement compared to maxillary premolar extraction resulted in an increase of the facial (N′-Prn-Pog’) and soft tissue profile (N′-Sn-Pog’) combined with an increase of the mentolabial angle (Pog’-B′-Li) that led in total to a decrease of the facial convexity. On the other hand, in Class III patients, maxillary advancement or bimaxillary surgery led to a decrease of the facial and soft tissue profile as well as mentolabial fold and thus, to an increase of the facial convexity. Camouflage therapy, whereas, tended to lead to an intensification of the underlying profile in both malocclusions. Notable statistical changes in the pronounced malocclusion occurred only in Class III patients comparing the maxillary advancement with the mandibular setback or bimaxillary surgery. Here, the maxillary advancement led to a significant reduction of the upper lip thickness (A–A’ on NL) as well as the distances of the upper and lower lip to the E-line.

It must be taken into account that these changes are likely to affect the attractiveness of the soft tissue profile. In this context, Ghorbanyjavadpour and Rakhshan investigated esthetic factors of the profile silhouettes from 70 Class I subjects by different cephalometric variables and found that improved profile esthetics will be caused by more convex skeletal and soft-tissue profiles, less prominent noses with higher tips, subnasales anterior to the upper lip, more protruded upper lips, less prominent lower lips, smaller interlabial gaps, and more protruding chins [31].. Previously, Khosravanifard et al. reported that especially an excessive mandibular retrusion made both men and women unattractive. Additionally, closeness of lips to the middle of vertical chin-nose distance enhanced female beauty only. Straight profiles and more protruded maxillae merely made men more attractive [8]. Against this background, the present data suggest an aesthetic improvement due to surgical intervention compared to camouflage treatment, but without taking into account the underlying gender. This confirms the results of Bou Wadi et al., who reported that the profile silhouette of predictive tracing simulating orthognathic surgery showed to be more attractive than that of Class III camouflage orthodontic treatment [20].

## Conclusion

Taking into account the limited significance of the findings due to the inhomogeneous group sizes, the current results indicate that weighing the therapeutic options within the moderate malocclusion seems to be of greater importance than in the case of pronounced malocclusions. Especially, if a camouflage treatment can be considered, patients as well as practitioners have to be aware of the possible influence on the soft tissue profile. Especially, the following soft tissue changes should be considered:

### Moderate class II treatment

- Upper premolar extraction vs mandibular advancement: Facial convexity decreases under orthognathic surgery, the facial profile (N′-Prn-Pog’), soft tissue profile (N′-Sn-Pog’), and mentolabial angle (Pog’-B′-Li) increases

### Moderate class III treatment

- Lower premolar extraction vs. maxillary advancement: Regarding N′-Prn-Pog’ and N′-Sn-Pog’ the facial convexity increases due to maxillary advancement and decreases slightly in the course of camouflage treatment. The distance of the lips to the E-Line decreases in the surgery group and increases in the extraction group, while after each therapy concept the mentolabial fold was less visible, with exception after orthognathic surgery where the fold became more prominent
- Lower premolar extraction vs. bimaxillary surgery: double jaw surgery tends to lead to similar changes as monomaxillary MxA, but less intensive, as N′-Prn-Pog’, Pog’-B′-Li and Ls-E-line decrease

### Pronounced class III treatment

- Maxillary advancement vs. mandibular setback or bimaxillary surgery: Upper lip thickness (A–A’ on NL) as well as the distances of the upper and lower lip to the E-line decrease significantly.

## Supplementary Information

**Additional file 1.**

## Data Availability

The data supporting the findings of this research can be obtained directly from the corresponding author.
