# Supplementary material for: Effects of different surgical techniques and displacement distances on the soft tissue profile via orthodontic-orthognathic treatment of class II and class III malocclusions
Source: Head Face Med. 2021 Apr 14;17:13. doi: 10.1186/s13005-021-00264-4 (PMC8048257; doi:10.1186/s13005-021-00264-4)
Supplement: Supplementary file 1 — Additional file 1. [file 13005_2021_264_MOESM1_ESM.docx]

Table S1: (Supplementary material): Mean values and standard deviation (SD) of the angular and linear changes of the hard and soft tissue profiles in Class II and III patients depending on the different surgical treatment concepts with corresponding p-values of the comparisons between the genders

|  | **Class II malocclusion** | | | | | |  |  | **Class III malocclusion** | | | | | | | | | | |  |
| --- | --- | --- | --- | --- | --- | --- | --- | --- | --- | --- | --- | --- | --- | --- | --- | --- | --- | --- | --- | --- |
|  | MxA | |  |  | MnS | |  |  | MxA/MnS | |  |  | MnA | |  |  | MxS/MnA | |  | |
|  | Men | Women | p-value |  | Men | Women | p-value |  | Men | Women | p-value |  | Men | Women | p-value |  | Men | Women | p-value | |
|  | N = 18 | N = 18 |  |  | N = 10 | N = 15 |  |  | N = 36 | N = 27 |  |  | N = 8 | N = 21 |  |  | N = 6 | N = 8 |  | |
| SNA (°) | 4.93 (2.42) | 5.48 (2.24) | n.s. |  | 0.15 (1.54) | 1.14 (1.80) | n.s. |  | 3.21 (3.15) | 3.19 (3.28) | n.s. |  | 1.24 (1.88) | - 0.15 (1.98) | n.s. |  | -2.20 (2.81) | 0.36 (3.16) | n.s. | |
| SNB (°) | -0.12 (1.40) | -0.25 (0.96) | n.s. |  | -2.77 (1.47) | -2.37 (2.17) | n.s. |  | -1.99 (4.42) | -1.09 (3.16) | n.s. |  | 1.24 (1.88) | -0.15 (1.98) | n.s. |  | -2.20 (2.81) | 0.36. (3.16) | n.s. | |
| WITS (mm) | 5.52 (2.56) | 5.78 (2.82) | n.s. |  | 6.18 (3.65) | 6.14 (2.59) | n.s. |  | 7.66 (4.01) | 6.37 (5.45) | n.s. |  | -3.33 (1.68) | -5.65 (2.53) | <0.05 |  | -4.30 (3.58) | -3.36 (2.27) | n.s. | |
| NL/NSL (°) | 0.61 (3.40) | 0.24 (2.89) | n.s. |  | 1.18 (2.46) | -1.18 (2.25) | n.s. |  | 0.79 (4.42) | 0.50 (4.27) | n.s. |  | -1.46 (1.61) | -0.10 (1.91) | n.s. |  | 1.58 (4.59) | -1.44 (4.23) | n.s. | |
| ML/NSL (°) | 0.06 (3.05) | 0.29 (1.55) | n.s. |  | -0.14 (1.66) | -0.32 (2.81) | n.s. |  | 0.22 (3.92) | -1.73 (3.78) | n.s. |  | 1.38 (2.35) | 0.82 (4.87) | n.s. |  | 2.58 (2.29) | 0.28 (2.33) | n.s. | |
| N-A-Pog (°) | 5.42 (6.90) | 1.92 (9.03) | n.s. |  | 1.84 (4.01) | 2.59 (5.89) | n.s. |  | 3.83 (7.71) | 2.57 (7.06) | n.s. |  | 2.63 (1.84) | 5.75 (5.38) | n.s. |  | 4.14 (2.32) | 4.44 (4.97) | n.s. | |
| N'-Prn-Pog' (°) | -3.32 (5.15) | -4.33 (4.71) | n.s. |  | -0.68 (6.63) | -4.36 (2.88) | n.s. |  | -5.62 (5.70) | -4.76 (6.03) | n.s. |  | 3.75 (2.57) | 4.55 (3.11) | n.s. |  | 3.38 (4.10) | 3.94 (5.81) | n.s. | |
| N'-Sn-Pog' (°) | -4.05 (6.08) | -7.42 (5.10) | n.s. |  | -2.44 (7.76) | -3.45 (3.82) | n.s. |  | -7.68 (6.32) | -3.96 (8.19) | <0.05 |  | 5.10 (2.71) | 4.70 (4.22) | n.s. |  | 5.22 (5.03) | 5.18 (5.60) | n.s. | |
| Cm-Sn-Ls (°) | 0.64 (7.19) | 4.01 (8.55) | n.s. |  | -0.26 (8.15) | 0.92 (5.23) | n.s. |  | 2.64 (10.80) | 0.38 (13.80) | n.s. |  | 1.04 (8.19) | 1.91 (8.44) | n.s. |  | 4.04 (12.50) | -1.89 (11.40) | n.s. | |
| Pog'-B-Li (°) | -10.10 (10.10) | -11.90 (14.2) | n.s. |  | -9.67 (12.10) | -11.50 (15.30) | n.s. |  | -11.70 (16.50) | -16.60 (18.30) | n.s. |  | 16.30 (9.63) | 22.10 (17.7) | n.s. |  | 11.30 (13.10) | 6.64 (14.10) | n.s. | |
| LiPog'-Gn'H (°) | -0.03 (10.0) | 9.63 (9.50) | <0.01 |  | 1.43 (9.01) | 12.60 (15.60) | n.s. |  | 3.08 (14.00) | 2.66 (17.30) | n.s. |  | -1.84 (12.50) | -7.51 (12.90) | n.s. |  | -26.10 (8.35) | -6.86 (20.50) | n.s. | |
| Sn-Sto (mm) | 1.69 (2.65) | 1.71 (1.95) | n.s. |  | 1.23 (3.57) | 0.21 (2.60) | n.s. |  | 2.00 (4.16) | 1.04 (2.82) | n.s. |  | -0.19 (2.14) | -0.46 (2.37) | n.s. |  | -3.58 (0.73 | -1.28 (1.68) | <0.01 | |
| A–A'/NL (mm) | -2.14 (2.54) | -2.08 (2.24) | n.s. |  | -0.45 (3.00) | -0.56 (1.75) | n.s. |  | -0.94 (3.33) | 0.03 (2.97) | n.s. |  | -0.44 (1.66) | 0.03 (2.23) | n.s. |  | 0.42 (1.95) | -0.689 (2.44) | n.s. | |
| Ls-E-Linie (mm) | 3.71 (3.66) | 3.59 (3.04) | n.s. |  | 1.82 (2.71) | 1.24 (2.23) | n.s. |  | 3.56 (4.04) | 3.49 (2.79) | n.s. |  | -2.44 (2.84) | -2.70 (1.99) | n.s. |  | -2.08 (2.84) | -3.05 (3.10) | n.s. | |
| Sto-Gn' (mm) | -3.70 (4.03) | -2.79 (4.47) | n.s. |  | -1.58 (6.94) | -1.67 (3.75) | n.s. |  | -1.60 (7.17) | -1.46 (5.32) | n.s. |  | 0.20 (5.28) | 0.19 (3.80) | n.s. |  | 1.52 (4.97) | -1.98 (4.06) | n.s. | |
| B–B'/ML (mm) | 0.75 (2.04) | 0.11 (1.78) | n.s. |  | 1.45 (2.75) | 1.29 (1.98) | n.s. |  | 0.98 (2.70) | 1.63 (2.26) | n.s. |  | 0.33 (1.60) | 0.30 (2.05) | n.s. |  | -0.48 (3.13) | -1.00 (0.943) | n.s. | |
| Li-E-Linie (mm) | 1.06 (1.53) | 1.28 (1.59) | n.s. |  | -0.38 (3.23) | 0.81 (3.06) | n.s. |  | 0.38 (3.60) | 0.17 (2.87) | n.s. |  | -0.24 (1.91) | -1.58 (3.08) | n.s. |  | -4.97 (5.17) | -2.50 (3.76) | n.s. | |
